# The Role of Teachers’ Emotions and Their Assessment in Professional Development Research: A Systematic Review

**DOI:** 10.1007/s10648-025-10048-w

**Published:** 2025-07-12

**Authors:** Mareike Ehlert, Carola Grunschel, Friederike Koehler

**Affiliations:** 1 University of Münster, Münster, Germany; 2 Centre of Excellence in Music, Mind, Body, and Brain, Department of Music, Art, and Culture Studies, University of Jyväskylä, Jyväskylä, Finland

**Keywords:** Educational change, Educational reform, Teacher learning, Feelings of change, Teacher education

## Abstract

Professional development (PD) both influences and is influenced by teachers’ emotions, yet emotions remain unaddressed in PD frameworks. This study pursues three objectives: (1) to offer a theoretical framework illustrating how PD processes influence and are influenced by teachers’ emotions, (2) to systematically review how empirical studies have assessed teacher emotions in PD, and (3) to synthesize recommendations on how teacher educators can attend to emotions in PD. The literature review identified 25 studies, most of which assessed emotions as secondary outcomes (e.g., general emotional experiences) without a consistent theoretical framework. Assessments predominantly relied on interviews and focused on emotions related to implementation objects or general job experiences. Emotions were typically captured as current or retrospective emotions, with limited focus on prospective emotions. Key recommendations included promoting emotional awareness, supportive environments, and collaboration. For future directions, we propose a more systematic approach to theorizing and measuring teacher emotions in PD.

## Introduction

Considerable emphasis is placed on the role of teachers in driving reforms within the educational system (Slavin, 2020). Professional development (PD) programs are commonly implemented alongside reforms to enhance teachers’ knowledge, beliefs, and practices, with the ultimate goal of PD achieving lasting improvements in student outcomes (Borko, 2004; Desimone, 2009). For example, the PD program implemented within the famous *Success for All* initiative, a comprehensive school reform model designed to improve reading outcomes in disadvantaged schools (Cheung et al., 2021), is known to be integral to the overall reform’s success.

While previous research has already summarized knowledge about the cognitive aspects that accompany PD processes, including factors like teacher knowledge and beliefs (e.g., Borko, 2004), emotions have often been overlooked in common frameworks of teacher learning (Desimone, 2009). However, as emotions play a pivotal role in predicting instructional change (Frenzel et al., 2021), investigating teachers’ emotions is essential for enhancing the impact of PD programs and, ultimately, driving educational reforms.

This study aims to advance research on teacher emotions in PD by pursuing three main objectives: First, we offer a theoretical integration of the role of emotions within PD processes by presenting a framework that illustrates how PD influences and is influenced by teachers’ emotions. Second, through a systematic literature review, we analyze how previous empirical studies have assessed teacher emotions in the context of PD, including (a) the emotion theories previous studies were based on, (b) the methods used to assess teacher emotions in PD processes, (c) the specific aspects within PD toward which teachers’ emotions are assessed (e.g., the implementation object, teacher PD experiences, or their job experiences), and (d) the point of time in the PD process to which the assessed emotions refer (e.g., prospective, current, or retrospective). As a third objective, we synthesize the recommendations by the reviewed studies regarding how teacher educators can attend to teacher emotions in PD. Understanding these aspects can provide valuable insights for researchers to ‘map the terrain’ of what we know and do not know as a field (Borko, 2004), as well as for practitioners to better address teachers’ emotions in PD programs (Chen, 2020).

### Teacher PD

Typically, PD programs involve structured opportunities for teachers to engage in learning activities aimed at improving instructional methods (Desimone, 2009). PD programs are thereby versatile, as they can include initial training as well as regular in-class coaching, feedback sessions, or collaborative team meetings. Moreover, they can address a wide range of topics (e.g., student-centered learning, differentiated instruction, or technology integration), accommodate teachers at various stages of their careers (e.g., from pre-service teachers to experienced in-service teachers), and may vary in their duration depending on the scope and objectives of the program (e.g., from a comprehensive curriculum reform to a short-term workshop; Desimone, 2009).

Desimone’s (2009) widely cited framework describes this multi-faceted nature of PD by conceptualizing PD as a process of four components. As a first component, the model identifies key features of effective PD, including a specific content focus, active learning opportunities, coherence with teachers’ needs, sustained duration, and collective participation of school staff. According to the framework, these features influence the second component, changes in teachers’ knowledge, skills, and attitudes. Changes in knowledge and attitudes can further impact teachers’ instructional practices (component 3) as well as student outcomes (component 4). Additionally, the model describes the importance of context characteristics, such as the school environment and leadership support, which underlie all four components. However, in this framework (Desimone, 2009), the role of emotions is lacking, even though emotions can powerfully shape teachers’ motivation, engagement, and adoption of instructional practices (e.g., Frenzel et al., 2021). Given their significant impact, it is essential to consider the role of emotions more closely in the context of PD.

### The Role of Teacher Emotions in PD

While there has been an ongoing controversial debate on a universal definition of emotions in general emotion research, most researchers agree on a multicomponent psychological state, characterized by dynamic changes in affective feelings (e.g., joy), cognition (e.g., “This PD is really helpful for teaching classes.”), action tendencies (e.g., the urge to implement new strategies), expression (e.g., positive facial expressions), and physiology (e.g., change in muscle tone; Paul et al., 2020). Positive emotions, such as joy, satisfaction, and pleasure, as well as negative emotions, such as anger, frustration, sadness, and anxiety, emerge as a result of individuals’ appraisals of situations (Sutton & Wheatley, 2003; Taxer & Frenzel, 2015). This means that emotions are reactions to the evaluation of a stimulus, whether internal (e.g., thoughts or memories) or external (e.g., interactions or events). Generally, emotions are seen as crucial for the human psyche (Gross & Barrett, 2011), motivating and influencing foundational processes in cognition and behavior in interaction with the environment (Izard, 2009).

Given their pervasive influence in everyday life, emotions are likely to also play a central role in PD contexts. PD contexts can trigger strong emotional reactions because they are, in essence, contexts of change: They introduce new concepts, require teachers to reflect critically on their practices, and challenge their existing beliefs (Van Veen & Sleegers, 2009). Following the broaden-and-build theory (Fredrickson, 2001), emotions are likely to act both as facilitators and inhibitors in PD: While positive emotions can expand a teacher’s thoughts and actions, negative emotions may narrow thinking and behavior.

#### Integrating Emotions in PD Processes

**Fig. 1:**
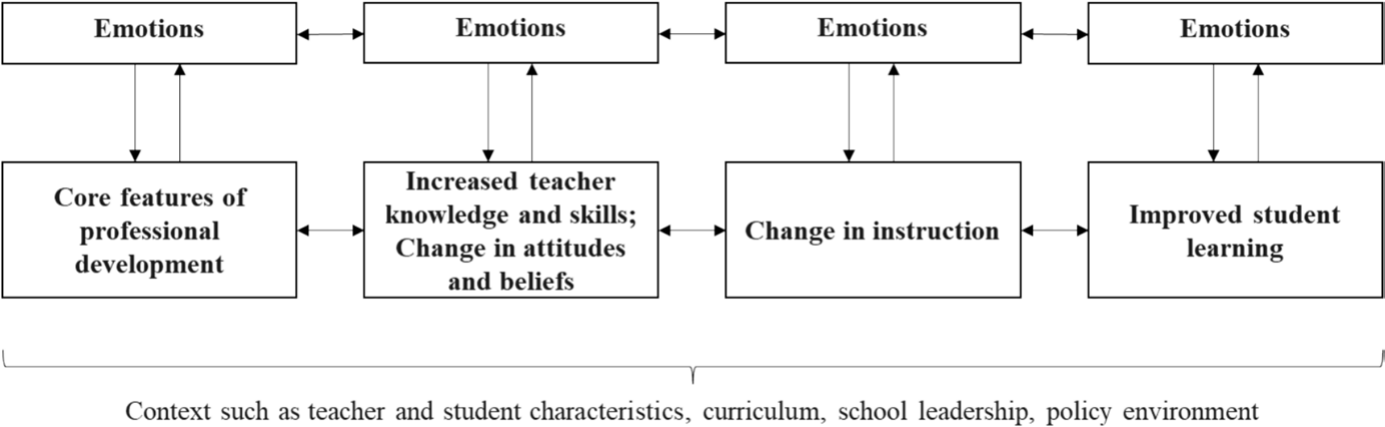
Adjusted theoretical framework (Desimone, 2009) on the role of teacher emotions in PD

Note. The four components of Desimone’s (2009) model are shown in the center of the figure. The figure illustrates that emotions can both influence (downward arrow) and be influenced by (upward arrow) each PD component. Moreover, emotions experienced in one component can shape emotional experiences in other PD components (reciprocal arrows between emotions).

Returning to established models of PD, emotions can be meaningfully integrated within Desimone’s (2009) framework (see Fig. 1). Specifically, emotions can both influence and be influenced by each component of the PD process. Moreover, as emotions are inherently dynamic, emotions experienced in relation to one component can shape emotions experienced in response to other components. To explain these mechanisms, we will first describe how emotions may interact with each individual PD component before exploring the reciprocal relationships between emotions across the PD process.

In the first component of the model (“Core features of PD”), emotions in PD can be influenced by the core features of PD, such as the content and structure of the program. For example, when the PD content aligns with a teacher’s existing beliefs, positive emotions like enthusiasm are more likely to arise. Similarly, different PD activities can evoke emotions. For instance, coaching formats may trigger positive emotions like excitement through collaborative learning. Conversely, the positive or negative emotions that teachers experience in PD may also shape their perceptions of the key features of PD. For example, a teacher attending a PD program on student-centered teaching methods who enjoys the topic is likely to perceive the program as meaningful and experience joy and optimism.

In the second component (“Increased knowledge and skills; Change in attitudes and beliefs”), emotions may influence the acquisition of knowledge and attitudes, as teacher emotions affect how concepts are internalized, retained, and applied (Seiz et al., 2015). While positive emotions ensure longer engagement with tasks, facilitating knowledge acquisition, negative emotions may lead to avoidance of unpleasant tasks. In the case of a PD program focused on student-centered learning, if a teacher feels overwhelmed by the demands of managing student-led discussions, they may develop negative attitudes towards this approach. Conversely, the acquisition of new knowledge and shifts in attitudes can also shape teachers’ emotional responses. For instance, a teacher who successfully understood the concepts of student-centered methods may experience positive emotions toward these strategies.

In the third component (“Change in instruction”), emotions may impact teachers’ implementation of new practices. For instance, if a teacher experiences pride when students actively engage in problem-solving activities, it may encourage their sustained use of student-centered methods. Vice versa, if the teacher initially encounters difficulties—such as classroom management challenges or students struggling with self-regulated learning—they may feel frustration, which could again lead to reluctance to continue using these strategies.

In the fourth component (“Improved student learning”), as teachers’ emotions can directly impact student outcomes, as well as indirectly via student–teacher relationships (e.g., Frenzel et al., 2021), it seems plausible that emotions may also influence the overall impact of the PD program on student outcomes. For example, a teacher who feels empowered after a PD program on student-centered learning may more strongly encourage students to take ownership of their learning. At the same time, seeing students succeed—such as seeing their increased engagement or critical thinking skills—may, in turn, evoke feelings of satisfaction and joy in teachers.

Beyond their immediate relation with each PD component, emotions are also interconnected across different components of the PD process (Fig. 1). This interconnectedness may be understood as a spillover effect, where emotions experienced in one component can shape emotions in other components. For instance, if a teacher faces frustration when struggling with classroom management during implementation (component 3), this emotion may carry over and negatively influence their emotions regarding the students’ improvement (component 4). Conversely, if teachers experience pride from seeing their students engage more in student-centered learning, they may feel more joy in using these methods in future lessons. In sum, the adjusted model illustrates that teachers’ emotions in PD are context-sensitive, as they influence and are influenced by each component of the PD process (Fig. 1). Moreover, emotions are time-sensitive, as those experienced in one component are interconnected with emotions in subsequent components.

### Assessment of Teacher Emotions in PD

Following on from our adjusted model, it is essential to examine how previous research has assessed teacher emotions in PD. Reflecting on the methods of assessment may highlight methodological limitations in the field and point to opportunities for developing context-sensitive, time-sensitive, and multidimensional assessments, which accurately reflect the role of emotions (Fig. 1).

#### Theoretical Frameworks for Assessing Teacher Emotions in PD

Given that a comprehensive synthesis of how emotion theories have been used in the PD context is lacking, our first goal for the systematic literature review is to examine the theoretical frameworks previous research has applied, as theories form the foundation for all assessments. Since emotion research encompasses a wide range of theoretical perspectives, multiple theories could be relevant for exploring teacher emotions in PD. For example, the control-value theory (Pekrun, 2006) could be useful in examining how teachers’ emotions emerge based on their appraisals of control and value across the four PD components (Fig. 1). Additionally, certain theories may be particularly relevant to explore the influence of emotions on specific PD components. For instance, as outlined above, the broaden-and-build theory (Fredrickson, 2001) may be particularly useful in examining how positive emotions broaden cognitive and behavioral repertoires. Such knowledge of which theoretical approaches previous studies have used could reveal specific areas of focus and gaps in the literature. In turn, this could inform actionable recommendations for the theoretical outline of future research on teacher emotions (e.g., concerning the suitability of certain emotion theories, or reliable and valid emotion measurements) and guide the practical design of PD programs (e.g., incorporating emotions as a content component).

#### Methods of Assessing Teacher Emotions in PD

Moreover, understanding how teacher emotions in PD have been measured is essential. Generally, emotions can be measured as a general trait, reflecting an individual’s typical emotional responses, and as states within specific contexts, where situational or contextual factors such as PD components influence the emotional experience. In quantitative research on teacher emotions, teacher emotions are frequently viewed as trait-like constructs, often measured using self-report tools like the Teacher Emotions Scales (TES; Frenzel et al., 2016) or the Teacher Emotion Questionnaire (TEQ; Burić et al., 2018). These tools could be adapted for studying emotions in PD by exploring how teachers’ emotions influence, for example, their adoption of new instructional strategies (cf. Figure 1, component 3). Moreover, experience sampling methods (e.g., Becker et al., 2015), short post-lesson or daily diary questionnaires (e.g., de Ruiter et al., 2020), or interviews (Hargreaves, 2000) are commonly employed to measure state-like emotions. These approaches may also be helpful in the context of teacher PD, as they allow for real-time tracking of emotions and can capture how emotions develop across PD components as well as how teachers respond to evolving challenges and learning experiences within PD programs. Despite these advances, much of the existing research has focused on teacher emotions experienced in the classroom (Frenzel et al., 2021), and most conceptual models have addressed teacher emotions in general (e.g., Chen, 2020; Fried et al., 2015). As a result, there remains a gap in understanding how emotions are measured in the PD context.

#### Object Focus of Emotions

Moreover, emotions are not isolated sensations, but always have a specific focus, meaning that they are tied to particular situational references (Shuman & Scherer, 2014). In the PD context, teachers’ emotions may be directed toward the *implementation object* itself, that is, the teaching methods, curricular reforms, or interventions introduced through the PD program (Teerling et al., 2018). For example, teachers might feel anxiety towards student-centered learning when first encountering it during PD (cf. Figure 1, component 1), frustration towards student-centered learning when acquiring more knowledge about the content (component 2), joy towards student-centered strategies as they successfully integrate them into their classroom teaching (component 3), or feel joy regarding student-centered learning as they see their impact on students’ critical thinking skills (component 4). Second, emotions might be directed towards their *PD experience* itself (Saunders, 2013). For example, teachers might feel frustration or disappointment with the PD program’s relevance or structure (component 1), experience anxiety or stress about not having acquired enough knowledge (component 2), feel proud of themselves for having learnt how to implement new strategies in the classroom (component 3), and pride towards their learning as they observe positive changes in student engagement and learning (component 4). Third, their emotions might center on general *job experiences,* such as the emotions teachers have regarding their overall job satisfaction (Day & Lee, 2011). In this regard, their emotional focus may be directed toward the context characteristics outlined in the adjusted model (Fig. 1). For instance, teachers may feel frustration toward their school leadership for a lack of support, or resentment towards policy decisions that fail to allocate sufficient financial resources.

#### Point of Time in PD When Teacher Emotions can be Assessed

As emotions are dynamic, they can fluctuate depending on when in the PD process they are assessed. For example, a teacher might report frustration at a given moment during PD, but reinterpret that experience when asked to recall it afterward. This time sensitivity highlights the importance of adopting a temporal perspective when assessing emotions. Emotions in PD may be assessed *retrospectively* (i.e., to reflect on a past outcome in PD), *currently* (i.e., to assess a current state in PD), as well as *prospectively* (i.e., to assess how a future PD outcome is imagined) (Baumgartner et al., 2008). Clearly differentiating between what teachers anticipate, experience, and remember in emotion assessment can help understand how emotions develop over time, as well as how they shape teachers’ cognitive engagement, openness to new ideas, and willingness to experiment with and sustain instructional changes at different time points in PD.

### Addressing Teacher Emotions in PD Programs

Finally, beyond examining how teacher emotions are assessed in the context of PD, as a third objective, we aim to summarize practical recommendations from previous research on how teacher educators may attend to teacher emotions in PD contexts (Chen, 2020; Day & Lee, 2011). Since emotions can emerge in response to all components of the PD process (cf. Figure 1), it appears important to consider strategies that help teachers consciously experience, reflect on, and articulate their emotions across all four components. For one, teachers who feel emotionally understood and supported are likely to be more motivated and engaged throughout the PD process (Chen, 2020). Thus, directly addressing emotions may enhance learning and implementation (Bahia et al., 2013). To facilitate this, concrete strategies such as reflective discussions, role-playing scenarios, and guided journals could help teachers verbalize and process their emotions in relation to PD content and implementation challenges. Moreover, to systematically support teachers in managing their emotions throughout the PD process, structured interventions such as emotion regulation training (Frenzel et al., 2021) could be integrated into PD processes. Such training might help teachers build resilience and patience across all four components, for instance, when they do not see immediate student progress (Fig. 1; component 4). However, given that these ideas are largely theoretical or based on single case studies, and a comprehensive, research-based taxonomy is not yet available, this study aims to consolidate research-based insights on effectively addressing the emotional needs of teachers in PD.

### Research Questions

Building on our first goal of introducing a framework for integrating teacher emotions into PD (Fig. 1), the second goal of this study is to systematically review how previous research has assessed teacher emotions within PD contexts. Specifically, we aim to answer the following research questions:

1. What theories of emotion form the foundation of studies on teachers’ emotions in PD processes?
2. What are the methods used to assess teachers’ emotions in PD processes?
3. What are the specific aspects within PD toward which teachers’ emotions are assessed (i.e., implementation object, the PD experiences, or general job experiences)?
4. To which point of time in the PD process (retrospective, current, or prospective) do the assessed emotions refer?

Additionally, to offer practical insights for teacher educators, the third goal of this study focuses on addressing the following research question in the systematic review:

(5) What recommendations do the studies provide for teacher educators to attend to teacher emotions in PD programs?

## Method

### Eligibility Criteria

The literature review was conducted in accordance with the Preferred Reporting Items for Systematic Reviews and Meta-Analyses (PRISMA) guidelines (Liberati et al., 2009). Criteria for inclusion were pre-specified according to the PICOS framework (participants, interventions, comparisons, outcomes, and study design) (Liberati et al., 2009) and can be seen in Table 1. Studies investigating teachers at any stage in their career (e.g., pre-service, in-service) were included. In defining our inclusion criteria, we opted to incorporate studies on pre-service teacher training alongside those focusing on in-service PD. This decision is grounded in the understanding that pre-service teacher training is generally recognized as the ‘first phase’ of PD (e.g., Blömeke, 2019; Darling-Hammond & Bransford, 2005). Including these studies thus provides a comprehensive view of the entire continuum of teacher education, from initial preparation through lifelong learning. The studies had to include and provide a description about PD (e.g., content, duration, and activities) but PD could encompass any subject and any duration. Consistent with prior conceptualizations of PD (Desimone, 2009), we deliberately adopted a broad definition of PD, encompassing initiatives of any subject matter, scope, and duration (e.g., ranging from extensive curriculum reforms to brief, targeted workshops). Outcomes were not specified in the search syntax in order to get a complete list of variables measured in this context. We also did not specify comparators and accepted various study designs to get a complete overview of the topic, but non-peer-reviewed articles were excluded. Studies had to report on the emotions experienced by teachers (and not students).

**Table 1:** Eligibility criteria according to the PICOS framework

Domain	Inclusion criteria
Participants	• Teachers at all stages in their career
Interventions	• Professional development activities designed for (pre-service) teachers (e.g., programs, teacher trainings, professional learning teams, summer schools, and workshops) on any subject and of any length
Comparators	• Not specified
Outcomes	• Not specified
Study designs	• Quantitative (e.g., experiment, intervention, and surveys) • Qualitative (e.g., interviews, focus groups, and case reports) • Mixed methods

### Search Strategy

The databases PsycInfo, PubPsych, ERIC, and ScienceDirect were used as electronic databases. Using Boolean operators, a variety of terms related to teachers (e.g., instructor, educator), PD (e.g., learning, change, training), and emotions (e.g., feel*, affect*) were employed. The syntax used for literature search in PsycInfo was:

(AB teacher OR AB instructor OR AB educator) AND (AB professional development OR AB learning OR AB change OR AB training) AND (AB emotion* OR AB feel* OR AB affect*).

Additionally, we used Citation Pearl Growing strategies in Google Scholar to search for relevant references of primary studies (Booth et al., 2021), and consulted artificial intelligence tools (i.e., elicit.org, researchrabbit.ai, and scite.ai) to expand our search. Only studies published in English or German were included. The literature search was conducted in June 2024.

### Study Selection and Analyses

We imported detected studies into CITAVI (Swiss Academic Software, 2022). Two researchers independently screened abstracts and rated them according to the eligibility criteria. In case of exclusion, the two raters reported the most prominent reason and resolved discrepancies by discussion. Afterwards, we read the full texts and, if in line with eligibility criteria, we coded the studies according to the PICOS categories (participants, intervention, comparator, outcomes, study design, and context), as well as according to categories relevant to the research questions (theory, assessment, focus, time of emotion, and suggestions for PD programs). Again, the two researchers rated independently and discussed discrepancies. To check for interrater reliability, Cohen’s kappa was calculated for each PICOS category as well as for each category relevant to the research question prior to the discussion of discrepancies (*M* = 0.89, Min = 0.73; Max = 1.00; Landis & Koch, 1977). The full results for how each individual study was finally coded in response to the research questions are provided in the supplemental material in the Open Science Framework under the following link: https://osf.io/rcm2s/. The supplemental material further contains information on the type of emotions assessed in the studies. Apart from a few studies evaluating specific emotions, most studies described assessments of general emotional experiences during the PD process.

## Results

### Studies

**Fig. 2:**
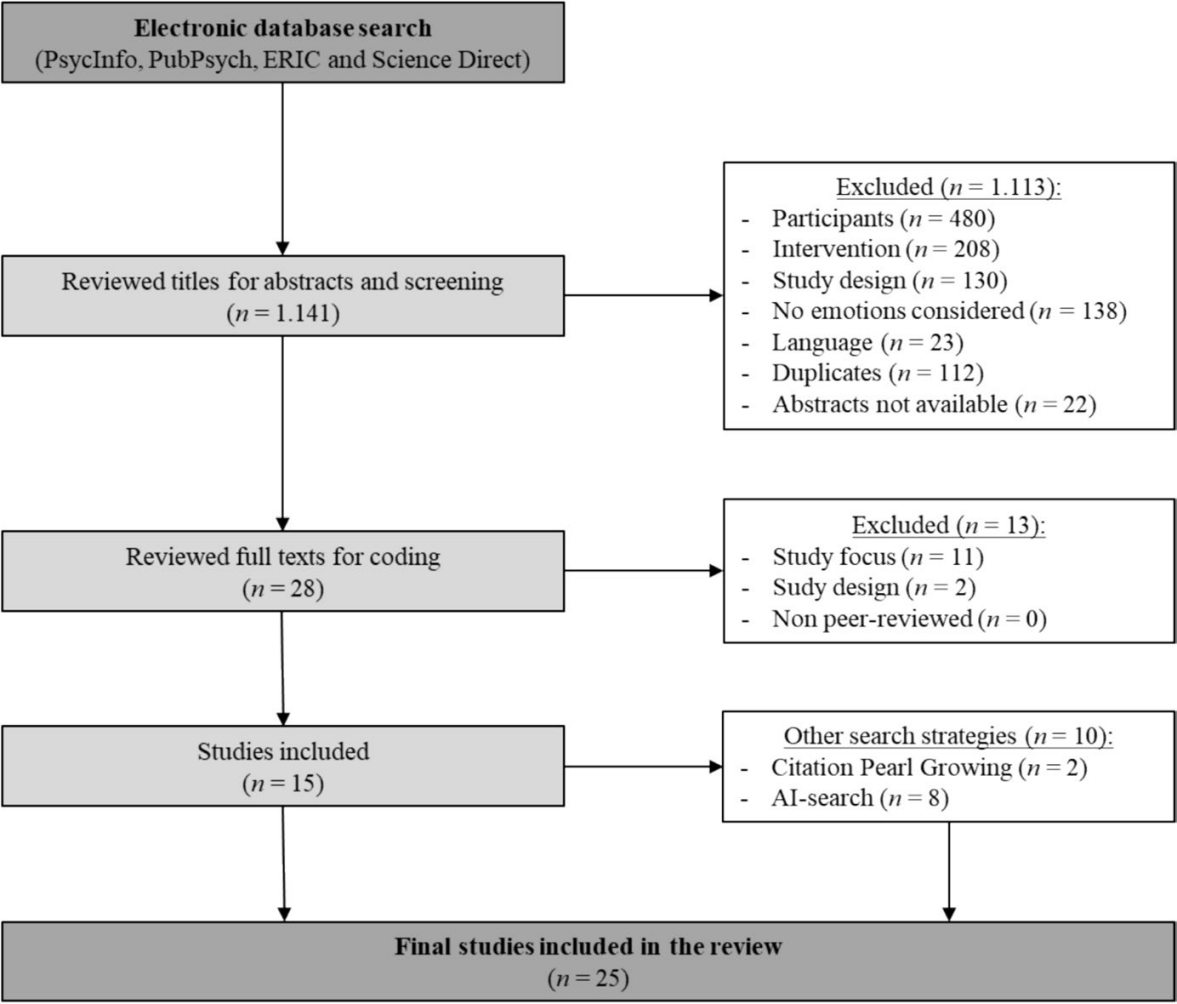
Flow diagram of study selection

The data base search according to our syntax yielded *n* = 1.141 studies (Fig. 2). Their abstracts were screened and *n* = 1.113 studies were removed for reasons listed in Table 1. The most common reasons for exclusion were that the participants were not teachers (*n* = 480), the study did not involve or reference a PD activity or program (*n* = 208), or the study design lacked data collection (*n* = 130). Full texts of the remaining* n* = 28 studies were assessed for eligibility. After that *n* = 25 studies remained for the narrative synthesis.

### Characteristics of Studies Investigating Emotions in PD Processes

The PICOS characteristics of studies investigating emotions in PD processes are displayed in Table 2. Most of the studies (*n* = 14) used a qualitative study design, followed by *n* = 6 quantitative and *n* = 5 mixed methods studies. Only one study (Bakkenes et al., 2010) had a comparator, while the other studies focused instead on the emotional responses elicited by the PD activities themselves. The PD programs varied significantly in content, duration, and activities. The duration, for example, ranged from short one-day workshops (e.g., Yoo & Carter, 2017) to extensive programs lasting several years (e.g., Darby, 2008; Saunders, 2013). Moreover, the content covered a wide range of topics, including classroom dialogue (Chang et al., 2018), comprehensive curriculum reform (Darby, 2008), science practices (Thomson & Turner, 2019), literacy coaching (Hunt, 2016), and teaching students with disabilities (Mauceri et al., 2012). Activities within these programs included reflective tasks, classroom observations, mentoring, collaborative projects, and more.

**Table 2:** PICOS characteristics of studies investigating emotions in PD processes

Reference	Participants	PD program intervention	Comparator	Outcomes/Main findings	Study design
Amory and Johnson (2023)	2 pre-service teachers in ESL	Content: first teaching experiences Duration: 16 weeks Activities: reflective teaching journal	-	Weekly meetings with the mentor and reflections provided a safe space to express emotions	Qualitative
Bakkenes et al. (2010)	94 secondary education teachers	Content: active and self-regulated student learning Duration: regular meetings over one year Activities: collaborative projects (EG1) and reciprocal peer-coaching (EG2)	Informal workplace learning	Both organized PD groups reported fewer negative emotions than teachers learning informally	Mixed methods
Chang et al. (2018)	6 secondary education teachers	Content: classroom dialogue Duration: one year containing three workshops Activities: reflections, video-based classroom observations	-	Teachers mostly displayed negative non-verbal emotional expressions during video-based PD exercises, but their intensity decreased over time	Quantitative
Darby (2008)	19 elementary school teachers	Content: comprehensive curriculum reform Duration: three years Activities: developing a school governance model, observations, co-teaching	-	Teachers first encountered fear and intimidation in the change process. Through the guidance of a literacy coach and university faculty, they were able to rebuild their self-perceptions, resulting in pride and excitement	Qualitative
Ehlert and Souvignier (2023)	43 elementary school teachers	Teachers did not receive the same PD, but reflected on PD experiences they received for a reading intervention to give recommendations for effective PD	-	Teachers recommended short, coherent PD with active learning opportunities. They explained this view through emotional reasons, i.e., that these features would reduce their anxiety and emotional exhaustion	Qualitative
Gaines et al. (2019)	10 middle school teachers	Content: use of student data Duration: four 45 min-sessions Activities: input phases, classroom observations	-	Teachers stated that pleasant emotions in PD facilitated implementation while unpleasant emotions in PD hindered implementation	Qualitative
Gallo (2016)	10 university language teachers	Content: language teaching Duration: four cycles of workshops over two years Activities: developing material and reflective practices	-	Teachers stated that their affective-emotional goals influenced their own learning more than instructional, occupational and developmental goals	Qualitative
Hascher and Hagenauer (2016)	117 pre-service teachers	Content: first teaching experiences during teaching practicum Duration: three to five weeks Activities: n/a	-	Student teachers mainly experienced their teaching practicum as emotionally positive. Self-efficacy positively predicted enjoyment in teaching practicum, and negatively predicted anxiety	Quantitative
Hunt (2016)	4 teachers	Content: literacy coaching interventions Duration: one year Activities: mentoring with literacy coaches	-	Teachers avoided emotions related to vulnerability (e.g., shame, guilt, and fear of failing)	Qualitative
Golombek and Doran (2014)	11 pre-service teachers	Content: Teaching English as a second language Duration: eight-week PD program Activities: reflective journal entries	-	Emotional experiences were prominent for teachers’ PD reflections. Student teachers connected their emotions to perceived teaching outcomes	Qualitative
Lemarchand-Chauvin (2023)	14 EFL pre-service teachers	Content: experienced emotions during teaching Duration: one to two weeks Activities: reflecting on video-taped classroom observations of their teaching	-	Reflecting on their classroom practices through the lens of emotions led to increased self-efficacy and professional growth. However, pre-service teachers showed limited emotional granularity, highlighting the need for further support in developing emotional awareness	Qualitative
Lemarchand-Chauvin and Tardieu (2018)	7 pre-service teachers	Content: first teaching experiences Duration: practical semester over one school year Activities: informal study diaries	-	Participants experienced negative emotions more often than positive emotions. Anger was mentioned most often	Qualitative
Mauceri et al. (2012)	61 pre-service special education teachers	Content: representations of disability Duration: n/a Activities: small-group PD with reflective discussions	-	PD helped increase emotional involvement and positive affect towards students with disabilities	Quantitative
Powell (1992)	15 modern languages teachers	Content: modern languages curriculum reform Duration: 20–30 h over seven months Activities: collaborative cross-country workshop in groups and a plenary feedback session	-	Teachers’ emotions in PD were dynamic over the course of seven months (enthusiasm, joy, nervousness, frustration, anger, guilt, anxiety) and tied to specific classroom experiences	Qualitative
Reinhold et al. (2021)	83 math teachers	Content: digital media in mathematics education Duration: four hours on one day Activities: hands-on activities and prepared material	-	Three different teacher orientations (nervous experts, confident advocates, and skeptical novices) emerged. PD helped reduce anxiety towards digital tools for nervous experts and skeptical novices	Quantitative
Saunders (2013)	27 teachers	Content: instructional practices Duration: twelve three-day seminars over four years Activities: discussions, reflections, observations and feedback	-	Emotions during teachers’ PD experiences were highly personal and developed in cyclical trends	Mixed methods
Scott and Sutton (2009)	50 elementary school teachers	Content: teaching writing Duration: eight workshops over four months Activities: observational learning, writing tasks	-	Teachers’ ratings of emotions towards writing became more positive over the course of PD, but returned to their initial levels after the program concluded. Qualitative interviews, however, showed that teachers simultaneously had mixed (both strongly negative and positive) emotions	Mixed methods
Šedová et al. (2017)	8 lower secondary teachers	Content: dialogic education Duration: one school year Activities: group discussions, video observations, reflective interviews	-	Teachers experienced many emotions during PD. Negative emotions promoted the implementation of novel practices	Qualitative
Teerling et al. (2018)	66 elementary school teachers	Content: reading instruction Duration: over half a year Activities: n/a	-	Frequency of cooperation, team communication, and team experiences predict affective-cognitive perceptions of the innovation	Quantitative
Thomson and Turner (2019)	67 teachers from various school types	Content: science practices Duration: six-week summer program Activities: inquiry-based PD in which teachers worked alongside scientists in a lab	-	Teachers’ emotions at the end of PD triggered changes in their science teaching practices. Positive emotions were associated with teachers’ gains in science knowledge	Mixed methods
Timostsuk and Ugaste (2010)	45 pre-service teachers	This study examined pre-service teachers’ development of professional identity during initial training at university, but after their teaching placement	-	Becoming a teacher is a highly emotional experience. Specifically, the level of negative emotions is high, as pre-service teachers emphasized failure or success rather than learning processes	Qualitative
Xu and Huang (2021)	9 secondary school teachers	Content: educational reform on a new admission test for high school students Duration: three years Activities: n/a	-	Teachers experience a decline in status, organizational support, and professional satisfaction during educational reforms, leading to negative emotions like stress and hopelessness. However, teacher training provides some relief by helping to adapt to the reform	Qualitative
Yoo and Carter (2017)	8 primary and secondary school teachers	Content: creative writing Duration: one day Activities: creative writing activities and reflections	-	Teachers experienced a wide range of emotions, both positive and negative, as they navigated conflicts between institutional demands and their intrinsic beliefs about effective teaching	Qualitative
Zwart et al. (2009)	28 secondary school teachers	Content: reciprocal peer coaching Duration: two-day workshop at the beginning and three follow-up meetings over one year Activities: hands-on activities, peer group discussions, coaching	-	Teachers felt pressure toward experimenting with new instructional methods, which decreased when they discussed their experiences during reciprocal peer coaching	Mixed methods
Zysberg and Maskit (2017)	133 teachers from various school types	Teachers did not receive the same PD, but were asked to indicate the PD stage they were in	-	Significant differences in the emotions experienced at work were found between PD stages. Enthusiastic and growth-oriented PD experiences throughout teachers’ career span were linked to positive emotions	Quantitative

### Results of Studies Investigating Emotions in PD Processes

Regarding the question of which emotion theories studies are based on (research question 1), *n* = 13 studies did not refer to any specific emotion theory (see Table 3). In contrast, four studies drew on the control-value theory (Pekrun, 2006). Additionally, two studies were based on the stages of concern (George et al., 2006), and another two studies utilized Vygotsky’s (1978, 1994) concept of the dialectical relationship between cognition and emotion, even though these two theories are not primarily conceptualized as theories of emotion.

**Table 3:** Results of studies investigating emotions in PD processes

What theories of emotion form the foundation of the study? (research question 1)	What are the methods used to assess teachers’ emotions in PD processes? (research question 2)	What are the specific aspects within PD toward which teachers’ emotions are assessed? (research question 3)	To which point of time in the PD process do the assessed emotions refer? (research question 4)
**None (*n* = 13)** (Bakkenes et al., 2010; Darby, 2008; Ehlert & Souvignier, 2023; Gallo, 2016; Hunt, 2016; Lemarchand-Chauvin & Tardieu, 2018; Mauceri et al., 2012; Powell, 1992; Scott & Sutton, 2009; Šedová et al., 2017; Timostsuk & Ugaste, 2010; Xu & Huang, 2021; Zwart et al., 2009; Zysberg & Maskit, 2017)	**Interviews (*n* = 13)** (Darby, 2008; Ehlert & Souvignier, 2023; Gaines et al., 2019; Gallo, 2016; Hunt, 2016; Lemarchand-Chauvin, 2023; Powell, 1992; Saunders, 2013; Scott & Sutton, 2009; Šedová et al., 2017; Thomson & Turner, 2019; Timostsuk & Ugaste, 2010; Xu & Huang, 2021)	**Job experiences (*n* = 8)** (Darby, 2008; Golombek & Doran, 2014; Hascher & Hagenauer, 2016; Lemarchand-Chauvin & Tardieu, 2018; Mauceri et al., 2012; Timostsuk & Ugaste, 2010; Xu & Huang, 2021; Zysberg & Maskit, 2017)	**Retrospective (*n* = 10)** (Amory & Johnson, 2023; Bakkenes et al., 2010; Darby, 2008; Ehlert & Souvignier, 2023; Hunt, 2016; Šedová et al., 2017; Teerling et al., 2018; Timostsuk & Ugaste, 2010; Xu & Huang, 2021; Zwart et al., 2009)
**Control-value theory (**Pekrun, 2006**) (*n* = 4)** (Chang et al., 2018; Gaines et al., 2019; Hascher & Hagenauer, 2016; Thomson & Turner, 2019)	**Reflective journals (*n* = 6)** (Amory & Johnson, 2023; Bakkenes et al., 2010; Golombek & Doran, 2014; Lemarchand-Chauvin & Tardieu, 2018; Xu & Huang, 2021; Zwart et al., 2009)	**Implementation object (*n* = 7)** (Bakkenes et al., 2010; Powell, 1992; Reinhold et al., 2021; Saunders, 2013; Scott & Sutton, 2009; Teerling et al., 2018; Zwart et al., 2009)	**Current (*n* = 8)** (Chang et al., 2018; Lemarchand-Chauvin, 2023; Lemarchand-Chauvin & Tardieu, 2018; Mauceri et al., 2012; Reinhold et al., 2021; Saunders, 2013; Yoo & Carter, 2017; Zysberg & Maskit, 2017)
**Stages of concern (**George et al., 2006**) (*n* = 2)** (Saunders, 2013; Teerling et al., 2018)	**Established scales (*n* = 6)** (Hascher & Hagenauer, 2016; Mauceri et al., 2012; Reinhold et al., 2021; Saunders, 2013; Teerling et al., 2018; Zysberg & Maskit, 2017)	**PD experiences (*n* = 5)** (Chang et al., 2018; Gaines et al., 2019; Gallo, 2016; Šedová et al., 2017; Thomson & Turner, 2019)	**A combination of time points (*n* = 5)** (Gallo, 2016; Golombek & Doran, 2014; Hascher & Hagenauer, 2016; Powell, 1992; Thomson & Turner, 2019)
Vygotsky (1978, 1994**) (*n* = 2)** (Amory & Johnson, 2023; Golombek & Doran, 2014)	**Self-developed scales (*n* = 4)** (Lemarchand-Chauvin & Tardieu, 2018; Powell, 1992; Scott & Sutton, 2009; Thomson & Turner, 2019)	**Both PD experiences and job experiences (*n* = 4)** (Amory & Johnson, 2023; Hunt, 2016; Lemarchand-Chauvin, 2023; Yoo & Carter, 2017)	**All three (*n* = 2)** (Gaines et al., 2019; Scott & Sutton, 2009)
**Other (each mentioned by *n* = 1 study)** *Achievement emotion theory* (Pekrun et al., 2007; mentioned by Chang et al., 2018), *Model of teacher emotions* (Frenzel 2014, mentioned by Hascher & Hagenauer, 2016), *Emotional Granularity* (Feldman-Barrett 2017, mentioned by Lemarchand-Chauvin, 2023) *Plutchik’s wheel of emotions* (Plutchik, 1980, mentioned by Lemarchand-Chauvin, 2023), *Emotional Orientations* (Hannula et al., 2016, mentioned by Reinhold et al., 2021), *Dualistic Approach of Emotions* (Sutton & Wheatley, 2003, mentioned by Yoo & Carter, 2017), *Interconnectivity of Emotions* (Parrot, 2001, mentioned by Yoo & Carter, 2017)	**(Video) observations (*n* = 2)** (Chang et al., 2018; Yoo & Carter, 2017)	**All three (*n* = 1)** (Ehlert & Souvignier, 2023)	**Prospective (*n* = 0)**

Regarding the methods used to assess emotions (research question 2), the majority of studies employed interviews (*n* = 13), often using open-ended questions (see Table 3). Six studies used reflective journals, which allowed teachers to reflect on their emotional experiences, while another six studies used established scales, such as the Achievement Emotions Questionnaire for Teachers (Frenzel et al., 2010) or the PANAS (Krohne et al., 1996). A smaller number of studies employed self-developed scales (*n* = 4) and (video) observations (*n* = 2) to evaluate emotions.

Concerning the focus of emotion assessment (research question 3), most studies either examined emotions related to the implementation object itself (*n* = 8), for example, teachers’ emotions regarding self-regulated student learning activities they had to implement (Bakkenes et al., 2010), or to general job experiences (*n* = 7), such as their general teaching experiences during teaching practicum (Hascher & Hagenauer, 2016). Five studies focused on teachers’ emotions regarding their own learning experiences during PD. Additionally, four studies addressed both PD experiences and job experiences. One study considered emotions in relation to implementation objects, job experiences, and PD experiences comprehensively (Ehlert & Souvignier, 2023).

The results related to the assessed time of emotions in PD processes (research question 4) reveal varied approaches across the studies (see Table 3). Ten studies examined teachers’ emotions retrospectively by remembering and reflecting on past experiences. Eight studies focused on current emotions that teachers experienced in the moment during the PD process, for instance, by including items such as “I generally feel tense and nervous while teaching in the practicum” (Hascher & Hagenauer, 2016). Moreover, five studies took a comprehensive approach by integrating both current and retrospective perspectives, and two studies even examined emotions across all three time points. No study focused exclusively on examining prospective emotions that teachers might anticipate feeling in their PD experience.

Table 4 provides an overview of the recommendations offered by the studies for addressing teacher emotions in PD (research question 5). Emotional awareness and reflection were emphasized by thirteen studies, and involved making emotions explicit (Gallo, 2016), asking for emotional expectations before PD starts (Thomson & Turner, 2019), and informing teachers that emotions are an integral part of the PD process (Saunders, 2013). Creating a supportive environment was recommended by eight studies. This included establishing a safe space where trust and respect are cultivated (Amory & Johnson, 2023), and where both negative and positive emotions are accepted (Šedová et al., 2017). Collaboration was recommended by seven studies and included using professional learning communities to voice and share emotions (Gaines et al., 2019), and reinforcing the idea that teachers are not alone in their emotional experiences (Amory & Johnson, 2023). Moreover, systematic activities were suggested by six studies, which included acting out scenarios to reduce fear (Ehlert & Souvignier, 2023) and implementing long-term PD programs to address emotional changes over time (Thomson & Turner, 2019). Finally, enhancing self-esteem was highlighted by four studies. Recommendations in this category included providing non-judgmental feedback (Darby, 2008), reinforcing value appraisals (Chang et al., 2018), and addressing the development of self-efficacy to alleviate anxiety (Hascher & Hagenauer, 2016).

**Table 4:** Recommendations by studies to address teacher emotions in PD processes

What recommendations do the studies provide for teacher educators to attend to teacher emotions in PD programs? (research question 5)
**Promoting emotional awareness and reflection (*n* = 13)** (Gallo, 2016; Golombek & Doran, 2014; Hascher & Hagenauer, 2016; Lemarchand-Chauvin, 2023; Lemarchand-Chauvin & Tardieu, 2018; Mauceri et al., 2012; Saunders, 2013; Scott & Sutton, 2009; Šedová et al., 2017; Thomson & Turner, 2019; Timostsuk & Ugaste, 2010; Zwart et al., 2009; Zysberg & Maskit, 2017)
**Creating a supportive environment (*n* = 8)** (Amory & Johnson, 2023; Chang et al., 2018; Hunt, 2016; Lemarchand-Chauvin, 2023; Saunders, 2013; Scott & Sutton, 2009; Xu & Huang, 2021; Zwart et al., 2009)
**Promoting collaboration between colleagues (*n* = 7)** (Amory & Johnson, 2023; Bakkenes et al., 2010; Darby, 2008; Gaines et al., 2019; Powell, 1992; Teerling et al., 2018; Yoo & Carter, 2017)
**Providing systematic PD activities (*n* = 6)** (Bakkenes et al., 2010; Ehlert & Souvignier, 2023; Lemarchand-Chauvin & Tardieu, 2018; Reinhold et al., 2021; Thomson & Turner, 2019; Timostsuk & Ugaste, 2010)
**Enhancing self-esteem (*n* = 4)** (Chang et al., 2018; Darby, 2008; Hascher & Hagenauer, 2016; Xu & Huang, 2021)

## Discussion

This study set out to advance research on teacher emotions in PD by pursuing three main objectives: First, we proposed an adjusted framework in the theoretical background to conceptualize how PD processes influence and are influenced by teachers’ emotions (Fig. 1). Building on this, our second goal was to systematically review how previous research has assessed teacher emotions in the context of PD processes. The empirical results of the reviewed studies thereby revealed several key trends in current studies assessing teachers’ emotions in PD processes: (1) a number of studies so far lack explicit theoretical frameworks on emotions (in PD), (2) their methods for assessing emotions are diverse, with a predominance of qualitative interviews, (3) the focus of emotional assessment primarily relates to implementation objects or job experiences, and (4) the temporal assessment of emotions reveals a strong emphasis on current and retrospective measurement, but little attention to prospective emotions. As a third objective, we sought to identify practical recommendations offered by the reviewed studies for teacher educators to attend to teacher emotions in PD. The studies revealed several constructive suggestions, which included emphasizing emotional awareness and reflection, creating a supportive environment, fostering collaboration, implementing systematic activities, and enhancing teachers’ self-esteem. Together, these findings offer important implications for both research and practice, suggesting the need for more theory-driven, context-, and time-sensitive approaches to assessing and addressing teacher emotions in PD.

### Emotion Theories

Regarding the theoretical frameworks (research question 1), a significant number of studies (13 out of 25) did not explicitly refer to any specific emotion theory. This lack of theoretical anchoring may suggest a more pragmatic or exploratory approach to understanding emotions in PD, in which emotions are often assessed as a by-product (e.g., Ehlert & Souvignier, 2023). Even when emotion theories were referenced, such as the control-value theory (Pekrun, 2006), or other theories such as the Stages of Concern (George et al., 2006), and Vygotsky’s (1978, 1994) sociocultural theory, the diversity of these theories highlights the absence of a unified, systematic framework that could serve as a foundation for building cumulative knowledge. This observation aligns with the lack of recognition of emotions in widely adopted teacher PD frameworks (Desimone, 2009), despite their substantial role and influence across all components of PD. Since a vast number of studies have investigated teacher emotions referring to emotions in the classroom (e.g., Thumvichit, 2024), future studies specifically focusing on teacher emotions during PD processes might build on conceptual models on general teacher emotions (e.g., Chen, 2020; Fried et al., 2015), or theoretical frameworks on general emotions, such as the control-value theory (Pekrun, 2006). In addition, theories that examine the impact of emotions on specific PD components (Fig. 1) could be informative. For instance, Fredrickson’s broaden-and-build theory (2001) may be particularly useful for examining how positive emotions enhance teachers’ knowledge acquisition and drive behavioral change in the classroom (components 2 and 3). Applying and testing existing emotion theories in the context of PD processes might be beneficial because these frameworks provide validated and reliable methods for a comprehensive understanding of emotions, enable comparisons across different studies and contexts, and ultimately help design emotionally supportive PD programs.

### Assessment Methods

Similarly, the methods used to assess emotions varied widely, with interviews being the predominant tool, employed in 13 studies (research question 2). This preference for interviews, especially those with open-ended questions, highlights the qualitative nature of the research on teachers’ emotions in PD processes, which was also visible in the PICOS categories. Other methods, such as reflective journals and standardized scales, were used in a smaller number of studies, generally reflecting an interest in both self-reflection and structured measures. However, the lack of standardized methods for assessing teacher emotions parallels the absence of a consistent theoretical framework, making it challenging to compare findings across studies. Future studies could employ widely used and validated scales on general emotions, such as the Positive and Negative Affect Schedule (PANAS; Krohne et al., 1996), holding the advantage of being widely disseminated, thus enabling comparisons both within PD studies and with other populations. Alternatively, researchers could transfer specific teacher emotion scales to the PD context, such as the Teacher Emotions Scales (TES; Frenzel et al., 2016) or the Teacher Emotion Questionnaire (TEQ; Burić et al., 2018). In addition, PD research might benefit from including both trait and state measures of emotions to control for general emotional dispositions. Furthermore, apart from self-report measures, physiological or observational assessments might contribute to a holistic understanding of teacher emotions in PD processes (Chang et al., 2018).

### Focus of Emotions

Regarding the specific PD aspect toward which emotions are assessed (research question 3), emotions were most commonly assessed in relation to either the implementation object (e.g., specific teaching practices such as writing) or to general job experiences concerning context characteristics (cf. Figure 1). This focus is indeed significant because understanding emotions tied to implementation objects, such as new teaching strategies or curricular changes, can provide insights into how these specific elements impact teachers’ willingness to adopt and sustain the practice (Bahia et al., 2013) in all components of PD (cf. Figure 1). Similarly, examining emotions directed at job-related experiences, such as workload, reflects important contextual factors that shape PD outcomes. However, since PD is inherently a process of teacher learning and growth (Borko, 2004), it is crucial to also consider the complex emotions teachers encounter related to the PD program itself and teachers’ own learning (Saunders, 2013). For example, teachers may experience anxiety about their own growth when confronted with the challenges of long-held beliefs (component 2), or feel pride in their own learning as they successfully implement new strategies in the classroom (component 3). Yet, these emotions toward the PD experience itself were rarely assessed in the reviewed studies. A more comprehensive approach to assessing emotions could include a focus on teachers’ experiences within the PD program, in addition to their reactions to specific implementation objects and general job experiences.

### Temporal Emotion Assessment

The findings related to the assessed time of emotions in PD processes (research question 4) showed that a significant number of studies concentrated on assessing emotions retrospectively after PD (e.g., Bakkenes et al., 2010). This approach allows for a deeper understanding of how teachers make sense of their emotional experiences in hindsight. Similarly, eight studies focused on emotions that teachers currently experience during PD (e.g., Chang et al., 2018). However, the absence of studies focused on prospective emotions suggests a gap in understanding how anticipated emotions might influence teachers’ future engagement and behavior in PD. For instance, a teacher might feel hopeful when envisioning how new techniques could enhance their classroom management and student engagement, or apprehensive about the time and effort required to integrate new methods into their established routine. In this regard, the broaden-and-build theory (Fredrickson, 2001) again offers a compelling framework for understanding emotions as a motivational motor in PD. Only two studies (Gaines et al., 2019; Scott & Sutton, 2009) explored emotions across all three temporal stages—current, retrospective, and prospective—indicating that a broader recognition of the interplay of emotions in PD could be helpful. Addressing this gap could provide a fuller understanding of how emotions influence teachers’ attitudes towards and engagement in PD, and of how emotions are interconnected with each other (Fig. 1).

### Recommendations

Finally, the recommendations for addressing teacher emotions in PD programs (research question 5) offer a valuable framework for both researchers designing interventions and teacher educators supporting teachers. The emphasis on emotional awareness, reflection, and creating supportive environments underscores a consensus on the importance of directly addressing emotions in PD (Frenzel et al., 2021). Key recommendations further included fostering collaboration through professional learning communities to facilitate shared experiences and collective emotional processing, as well as implementing systematic PD activities that proactively address emotional challenges. These recommendations offer practical guidance for integrating emotions into the four core components of PD (Desimone, 2009; Fig. 1). For instance, incorporating emotional check-ins at the start of PD sessions can create a supportive atmosphere and enhance teachers’ readiness to learn (Fig. 1; component 1). Emotion journals, used alongside teachers’ evolving beliefs or knowledge, may help participants reflect on and process their feelings throughout the program, and group reflections may particularly foster emotional engagement, self-awareness, and collaboration, supporting the transition to instructional changes (components 2 and 3). Moreover, boosting teachers’ self-esteem and recognition of their value in education, as posed by the control-value theory (Pekrun, 2006), is likely to be essential across all PD components. Specifically, this may include helping teachers develop confidence through non-judgmental feedback. Structured interventions were not part of the recommendations provided by previous research. However, emotion regulation training (Frenzel et al., 2021) could still be helpful in equipping teachers with strategies to manage emotions effectively throughout the entire PD process, specifically when challenges arise in instructional change (component 3) or when observing student learning outcomes (component 4). In sum, these recommendations offer a first practical framework for creating more emotionally supportive PD environments.

### Limitations

Since the wide range of methodologies and assessment tools used in the reviewed studies hinders direct comparisons, this review cannot provide a meta-analysis to quantify the influence of teacher emotions on PD effectiveness. For the same reason, the identified studies are not useful in identifying antecedents and consequences of emotions in PD processes. Moreover, it was not possible to specifically classify the studies according to the components in the model (Fig. 1), as the studies were often too vague or broad in their context, making it challenging to map them accurately to the framework. It is also not clear, for example, which strategies are most effective for addressing teacher emotions in PD, leaving open questions about the overall efficacy of the proposed recommendations. Furthermore, the quality of the included studies might have affected our findings, particularly regarding very small sample sizes, lack of comparators, and geographical limitations, which might have impaired the reliability and validity of the studies’ conclusions. Future research could include studies which test the effect of emotions on teachers’ learning development, and vice versa. In addition, there might have been confounding variables the review could not consider, including contextual factors such as school environment, cultural differences, and teacher characteristics (Desimone, 2009). Correspondingly, the identified recommendations’ applicability may vary depending on the specific context and type of PD program.

### Conclusion and Future Directions

In sum, our findings suggest that while there is a growing recognition of the importance of teacher emotions in PD, the field is still in a relatively nascent stage with diverse approaches and methodologies. Our results underscore the importance of integrating emotions into PD (Darby, 2008), and future research should invest in developing a more systematic theoretical approach as well as more unified methods for assessing teacher emotions in PD contexts (Chen, 2020). Specifically, we propose the following future directions for theorizing and assessing emotions in PD:

- *We need to link emotions to PD contexts:* Future studies could generally build on the framework presented in this paper (Fig. 1) to systematically examine how the core components of PD influence, and are influenced by, teachers’ emotional experiences. Studies should thereby clearly specify to which PD component the assessed emotions refer. As emotions are both context- and time-sensitive, their effects may differ depending on whether they emerge during the perception of core features (component 1), knowledge and belief development (component 2), instructional implementation (component 3), or the reflection on student outcomes (component 4).
- *We need to develop component-specific theories*: For each PD component, the adoption of specific theoretical approaches (e.g., Fredrickson, 2001) could be helpful in understanding the precise role and impact of emotions for different components in the PD process.
- *We need methodological consistency in the assessment of emotions:* To enable cross-study comparisons and cumulate knowledge, researchers should adopt validated tools and develop instruments that align with the framework’s individual components, as well as clearly distinguish whether trait or state emotions are being measured.
- *We need to assess and report emotional reference points:* To build knowledge of teacher emotions in PD, future research should systematically assess and report the distinct emotional reference points (e.g., emotions related to PD, implementation tasks, or the job context).
- *We need to assess and report the timing of emotions:* Future studies should assess and report the timing of emotions and explore in longitudinal designs how emotions evolve across different PD components. Differentiating between what teachers anticipate, experience, and remember is essential for understanding emotional trajectories, as well as for understanding the distinct effects of emotions at different temporal stages of PD.
- *We need to evaluate emotion regulation interventions:* Intervention studies could test strategies aimed at addressing teacher emotions during PD, such as reflective practices, emotional awareness training, and collaboration-based PD formats that help to regulate emotions in PD.

Thus, much work remains to be done, but the relevance of this research is clear: understanding the role of emotions in PD holds significant potential to drive educational change (Borko, 2004).

## Supplementary Information

Supplemental material is available in the Open Science Framework via the following anonymous link: https://osf.io/rcm2s/.
